# Anti-inflammatory Diets for Chronic Pain Disorders With a Focus on Fibromyalgia: A Narrative Review

**DOI:** 10.7759/cureus.112395

**Published:** 2026-07-10

**Authors:** Divya Priya, Prashanth Shetty, Geetha B Shetty

**Affiliations:** 1 Diet and Nutrition, Sri Dharmasthala Manjunatheshwara (SDM) College of Naturopathy and Yogic Sciences, Ujire, IND

**Keywords:** anti-inflammatory diet, chronic pain, fibromyalgia, gut microbiota, neuroinflammation, oxidative stress

## Abstract

Fibromyalgia (FM) is a chronic pain syndrome characterised by widespread musculoskeletal pain accompanied by fatigue, cognitive dysfunction ("fibro fog"), and sleep disturbances. Although traditionally regarded as a non-inflammatory condition, emerging evidence suggests that low-grade inflammation, oxidative stress, autonomic nervous system dysregulation, neuroinflammation, and alterations in the gut microbiota contribute to its pathophysiology. Consequently, anti-inflammatory dietary approaches are increasingly being investigated as adjunctive interventions for the management of FM.

This narrative review summarises the effects of dietary patterns, including Mediterranean, plant-based, low-FODMAP (Fermentable Oligo-, Di-, Mono-saccharides, and Polyols), elimination, and ketogenic diets, as well as nutritional supplements such as omega-3 fatty acids and antioxidants, on FM symptoms. Proposed mechanisms include modulation of pro-inflammatory cytokines, attenuation of neuroinflammation, improvement in mitochondrial function, and regulation of the gut microbiota. Although existing findings are promising, the current evidence remains limited by small sample sizes and methodological heterogeneity. Further large-scale, well-designed clinical trials are required to establish the efficacy of dietary interventions in the management of FM.

## Introduction and background

Fibromyalgia (FM) is a chronic pain disorder with a globally variable prevalence ranging from 0.2% to 6.6%, with pooled estimates suggesting a prevalence of approximately 2%-3% [1,2]. It occurs more commonly in women than in men, particularly among individuals aged 20-55 years [1]. Prevalence varies across geographic regions and clinical settings, with higher rates frequently reported in urban populations. In India, data from Lucknow demonstrated prevalence rates of 41.2 and 32.4 per 1,000 individuals in urban and rural populations, respectively [3]. Clinically, FM is characterised by widespread musculoskeletal pain, fatigue, sleep disturbances, and cognitive dysfunction, commonly referred to as "fibro fog" [4]. The pathophysiology of FM is multifactorial, with central sensitisation, neuroimmune alterations, autonomic dysfunction, oxidative stress, and mitochondrial impairment all implicated in disease development [5,6]. Beyond its clinical manifestations, FM places a substantial burden on affected individuals because of persistent pain, fatigue, sleep disturbances, and impaired daily functioning. Despite increasing awareness of the condition, variations in diagnostic criteria and frequent underdiagnosis suggest that the true burden of FM remains underestimated [1,2].

FM has long been regarded as a non-inflammatory disorder, largely due to the absence of the overt tissue damage typically observed in other rheumatological conditions [7,8]. However, this perspective is gradually evolving. The discrepancy between the severity of symptoms and the lack of evident peripheral pathology suggests the involvement of altered pain processing within the central nervous system [7]. Neuroimaging studies have demonstrated altered activity in key brain regions associated with pain perception, including the thalamus, insula, and anterior cingulate cortex, thereby supporting the concept of central sensitisation [7,9]. Furthermore, emerging biomarker evidence indicates the presence of low-grade systemic inflammation in FM, with elevated levels of pro-inflammatory cytokines such as interleukin-6 (IL-6) and interleukin-8 (IL-8) reported in affected individuals [10].

Diet plays an important role in regulating inflammatory pathways by modulating the production of pro-inflammatory cytokines, influencing gut microbiota composition, reducing oxidative stress, and improving metabolic homeostasis, thereby contributing to immune regulation [11-13]. In addition, nutritional supplements such as omega-3 fatty acids, polyphenols, and coenzyme Q10 (CoQ10) have demonstrated potential benefits in the management of chronic pain and fatigue [14].

Therefore, this narrative review aims to synthesise the available evidence regarding the relationship between anti-inflammatory dietary approaches and chronic pain, with a particular focus on FM. It further examines the proposed mechanisms, clinical evidence, current limitations, and implications for future research and clinical practice.

## Review

This narrative review was based on a literature search of the PubMed, Scopus, and Google Scholar databases. Peer-reviewed articles published between January 2000 and July 2025 were considered to provide a comprehensive overview of the available evidence on dietary interventions in FM. Search terms included combinations of "fibromyalgia," "anti-inflammatory diet," "Mediterranean diet," "plant-based diet," "low-FODMAP diet," "ketogenic diet," "gut microbiota," "nutritional supplements," and "chronic pain." Search strategies were adapted to the indexing systems and search functionalities of each database to maximise retrieval of relevant literature. Relevant articles were identified through database searches, and the reference lists of pertinent publications were examined to identify additional studies.

Eligible literature included original research articles, randomised and non-randomised clinical trials, observational studies, systematic reviews, meta-analyses, and narrative reviews addressing dietary and nutritional interventions in FM. Review articles were used to provide background information, summarise the existing evidence, and identify relevant primary studies, while conclusions regarding the effectiveness of dietary interventions were preferentially based on original clinical and observational studies whenever available.

Studies unrelated to FM, evaluating non-dietary interventions, published in languages other than English, presented only as conference abstracts without sufficient methodological information, or lacking adequate methodological detail were excluded. Studies were selected according to their relevance, scientific quality, and contribution to the objectives and scope of the review, to provide a balanced and comprehensive synthesis of the current evidence.

Pathophysiology of FM and potential dietary targets

Central Sensitisation

Central sensitisation is recognised as a fundamental mechanism underlying FM and is characterised by increased excitability of the central nervous system, resulting in impaired pain inhibition [15]. Patients with FM have demonstrated reduced pain thresholds, enhanced temporal summation, and exaggerated pain responses, reflecting abnormal amplification of central pain processing [15]. Neuroimaging studies further support this mechanism by demonstrating increased activation in pain-processing regions, including the thalamus, insula, and anterior cingulate cortex, even in response to low-intensity stimuli [15].

At the molecular level, central sensitisation is thought to result from enhanced excitatory neurotransmission, particularly involving glutamate and N-methyl-D-aspartate (NMDA) receptor-mediated signalling, leading to persistent neuronal depolarisation and sustained pain transmission [16]. Elevated glutamate concentrations in the cerebrospinal fluid and brain tissue of patients with FM, together with increased levels of substance P and altered blood-brain barrier permeability, may contribute to heightened pain sensitivity and central pain amplification [16].

These findings highlight the complex interplay between neural and biochemical mechanisms in the pathophysiology of FM. Dietary factors may also influence these pathways through the anti-inflammatory and antioxidant effects of polyphenols, omega-3 fatty acids, and other bioactive nutrients. Magnesium may reduce neuronal hyperexcitability by modulating NMDA receptor activity, while omega-3 fatty acids may support glutamatergic homeostasis by improving neuronal membrane function and glutamate transport. Together, these mechanisms may contribute to the modulation of neuroinflammatory processes and central sensitisation in FM, although direct clinical evidence remains limited [16-19].

Neuroinflammation

Neuroinflammation has emerged as a significant contributor to the pathophysiology of FM, with growing evidence suggesting dysregulation of neuroimmune responses within the central nervous system. Positron emission tomography (PET) imaging studies have demonstrated glial cell activation in patients with FM, while elevated levels of chemokines such as IL-8 indicate enhanced interactions between neurons and glial cells, contributing to pain sensitisation [20].

Activated microglial cells in patients with FM release pro-inflammatory cytokines, including tumour necrosis factor-alpha (TNF-α) and interleukin-1 beta (IL-1β), which contribute significantly to pain amplification and neuroinflammatory signalling [20]. However, PET imaging findings should be interpreted cautiously, as they provide indirect evidence of glial activation and require confirmation through further studies [20]. In addition, cytokines such as IL-1, IL-6, and TNF-α are known to influence pain-processing pathways and may play a role in sustaining central sensitisation and chronic pain in FM [21].

Collectively, emerging evidence suggests that neuroinflammation plays a significant role in the persistence and progression of FM. Consequently, anti-inflammatory dietary approaches rich in polyphenols, omega-3 fatty acids, and other plant-derived antioxidants may help modulate these neuroinflammatory pathways and potentially alleviate symptom severity [20-22].

Oxidative Stress and Mitochondrial Dysfunction

FM has been closely associated with oxidative stress and mitochondrial dysfunction, characterised by mitochondrial structural abnormalities, reduced enzymatic activity, and impaired ATP production [23]. Elevated levels of reactive oxygen species and oxidative damage are thought to contribute to pain, fatigue, and inflammatory processes in patients with FM [23].

Although evidence in patients with FM remains limited, nutritional interventions have shown potential to mitigate these pathological changes. Antioxidants such as vitamins C and E, along with polyphenols, may reduce oxidative damage, while magnesium and CoQ10 may support mitochondrial function. CoQ10 plays a key role in mitochondrial ATP synthesis and also functions as a potent antioxidant, potentially helping to alleviate oxidative stress-related symptoms in patients with FM [17].

Autonomic Nervous System Dysregulation

Autonomic dysfunction is increasingly recognised as an important feature of FM. Studies assessing heart rate variability have demonstrated increased sympathetic nervous system activity alongside reduced parasympathetic (vagal) activity, suggesting a disturbance in autonomic regulation [24,25]. Heart rate variability is a non-invasive measure of autonomic nervous system function and has been widely used to assess autonomic dysfunction in FM. Persistent sympathovagal imbalance may contribute to pain, fatigue, sleep disturbances, and altered stress responses commonly observed in patients with FM [24,25].

Alterations in Gut Microbiota

Recent studies suggest a potential association between alterations in the gut microbiota and FM, highlighting a possible role of the gut-brain axis in its pathophysiology [26]. The gut-brain axis represents a bidirectional communication network between the central nervous system and the gut microbiota, involving immune, neural, and metabolic pathways that may influence pain perception and symptom severity in patients with FM [26].

Microbial metabolites, particularly short-chain fatty acids such as butyrate, may help maintain intestinal barrier integrity and modulate immune responses, thereby potentially influencing neuroinflammatory processes and gut-brain communication [27]. Dysbiosis in gut microbiota may contribute to the pathogenesis of FM through mechanisms involving enhanced immune activation, systemic inflammation, and increased intestinal permeability, commonly referred to as leaky gut syndrome, which may further exacerbate pain signalling and neuroinflammation [27].

Anti-inflammatory diets and their impact on FM

Mediterranean Diet (MD)

The MD, characterised by a high intake of fruits, vegetables, whole grains, legumes, nuts, olive oil, and fish, is widely recognised for its anti-inflammatory and health-promoting properties. Adherence to the MD may reduce inflammation by lowering the production of pro-inflammatory cytokines, improving immune and metabolic function, and enhancing antioxidant defences. Olive oil, a major source of monounsaturated fatty acids and polyphenols, together with omega-3 fatty acids from fish, may further contribute to these effects by modulating inflammatory signalling pathways and reducing oxidative stress [28].

Although FM-specific evidence remains limited, studies involving musculoskeletal disorders suggest that the MD may provide beneficial effects on pain and inflammation [29]. Given that FM is associated with inflammation, oxidative stress, and immune dysregulation, improving dietary quality through adherence to the MD may help alleviate symptoms and improve quality of life. Furthermore, increasing evidence implicates both the gut and systemic microbiome in the pathophysiology of FM, highlighting a potential role for dietary interventions in modulating these mechanisms [30].

Plant-Based Diets

Plant-based dietary patterns, including vegetarian and vegan diets, are rich in fibre, antioxidants, and phytochemicals with recognised anti-inflammatory and antioxidant properties. Their high fibre content may promote gut microbiome diversity and the production of beneficial microbial metabolites, while their low saturated fat content may help reduce systemic inflammation. In addition, antioxidants such as vitamins C and E, carotenoids, and polyphenols may reduce oxidative stress, collectively supporting improved metabolic and immunological balance in patients with FM [31].

In a non-randomised controlled trial involving 18 patients with FM, a three-month vegan dietary intervention resulted in significant improvements in pain, joint stiffness, sleep quality, and overall health status [32]. Similarly, a systematic review of dietary interventions in FM, which included multiple clinical studies, reported improvements in patient-reported outcomes such as pain, fatigue, quality of life, and psychological well-being across several dietary approaches, including vegetarian diets; however, the overall quality of evidence was considered low [33].

Overall, plant-based dietary interventions appear to offer potential benefits in the management of FM, although further well-designed studies are required to clarify their long-term efficacy and underlying mechanisms [31,33].

*Low-FODMAP (Fermentable Oligo-, Di-, Mono-Saccharides, and Polyols)* *Diet*

The low-FODMAP diet, which restricts poorly absorbed short-chain carbohydrates, was initially developed for the management of irritable bowel syndrome (IBS). Interest in its application to FM has increased because of the high prevalence of gastrointestinal comorbidities among patients with FM [34,35]. A substantial proportion of individuals with FM experience IBS-like symptoms, suggesting a potential link involving the gut-brain axis and central sensitisation mechanisms [35].

Reduction of dietary FODMAP intake may therefore alleviate gastrointestinal symptoms in patients with FM and indirectly influence overall symptom severity [29]. Although current evidence remains preliminary, pilot studies have suggested that low-FODMAP interventions may improve not only gastrointestinal symptoms but also certain aspects of daily functioning and quality of life in patients with FM. Nevertheless, further large-scale studies are required to confirm these findings [33-35].

Because the low-FODMAP diet is a restrictive dietary approach, prolonged adherence without appropriate guidance may reduce dietary diversity and increase the risk of nutritional inadequacy. Therefore, it should ideally be implemented under the supervision of a registered dietitian. Following symptom improvement, foods should be systematically reintroduced to identify individual trigger foods and establish a personalised long-term dietary pattern rather than maintaining prolonged dietary restriction [36].

Elimination Diets and Food Sensitivities

Patients with FM have frequently reported worsening of symptoms following the consumption of certain food items, leading to increasing interest in the use of elimination diets in FM management. Observational studies have shown that many individuals with FM modify their dietary habits after diagnosis, commonly avoiding foods such as gluten and lactose in an attempt to reduce symptom severity [37].

Several clinical studies have evaluated dietary interventions, including gluten-free and low-FODMAP diets, in patients with FM. A systematic review reported that these dietary approaches were associated with improvements in symptoms such as pain, fatigue, and sleep disturbances. However, the overall quality of the available evidence remains limited because of methodological heterogeneity and small sample sizes [33].

Although elimination diets have demonstrated potential benefits in some patients with FM, dietary modifications should be individualised to minimise unnecessary dietary restrictions and maintain nutritional adequacy. Given the limited and heterogeneous evidence, their overall effectiveness remains uncertain and requires further investigation through well-designed clinical studies [33,37]. 

Ketogenic Diets

Nutritional ketosis induced by a ketogenic diet leads to the production of ketone bodies, including β-hydroxybutyrate (BHB). The anti-inflammatory effects associated with ketogenic diets have been attributed, in part, to the ability of BHB to inhibit activation of the NLRP3 inflammasome, thereby reducing the secretion of pro-inflammatory cytokines and attenuating inflammatory responses [18].

Although robust scientific evidence remains limited, emerging studies suggest that ketogenic diet-induced ketosis may offer potential benefits in FM. A pilot study evaluating a very low-calorie ketogenic diet demonstrated significant improvements in disease severity, quality of life, mood, and sleep among patients with FM, independent of weight loss. These findings suggest that ketosis may influence FM symptomatology through metabolic and neurobiological mechanisms [19].

Despite these promising findings, adherence to ketogenic diets may be challenging because of their restrictive nature, potentially affecting long-term compliance. In addition, prolonged adherence may increase the risk of nutrient inadequacy if dietary intake is not carefully planned. Although short-term studies have reported acceptable safety and tolerability, the long-term safety and sustainability of ketogenic diets in patients with FM remain uncertain and warrant further investigation [19]. The major dietary approaches and their proposed effects in FM are summarised in Table 1.

**Table 1:** Overview of dietary approaches in fibromyalgia FM: Fibromyalgia; FODMAP: Fermentable oligosaccharides, disaccharides, monosaccharides, and polyols; NLRP3: NOD-like receptor family pyrin domain containing 3

Dietary approach	How it may work	Key studies	What studies suggest	Key limitations
Mediterranean Diet	May reduce inflammation, improve metabolic and immune function, and support gut health	Koelman et al. [28] (systematic review & meta-analysis); Casini et al. [29] (randomised controlled trial; n = 100); Carrasco-Querol et al. [30] (pragmatic randomised clinical trial; n = 158)	Associated with reduced pain and improved quality of life	Limited fibromyalgia-specific trials; some evidence extrapolated
Plant-Based Diets	High in fibre and antioxidants, helping reduce oxidative stress and inflammation	Kaartinen et al. [32] (non-randomised controlled trial; n=18); Nadal-Nicolás et al. [31] (systematic review); Silva et al. [33] (systematic review)	Improvements in pain, sleep, and general well-being were reported	Evidence quality is low; lack of large randomised trials
Low-FODMAP Diet	Improves gut symptoms and may influence the gut–brain axis	Marum et al. [34] (pilot longitudinal interventional study; n = 38); Rodríguez-Castillejo et al. [35] (scoping review); Silva et al. [12] (randomised controlled trial; n = 46)	Reduced gastrointestinal symptoms and some functional improvement	Small sample sizes; limited impact on core FM symptoms
Elimination Diets	Identifies and removes potential dietary triggers	Almirall et al. [37] (systematic review); Silva et al. [33] (systematic review)	Some symptom relief reported (pain, fatigue, sleep)	Heterogeneous methods; inconsistent findings
Ketogenic Diet	Ketone bodies may reduce inflammation via NLRP3 inhibition	Youm et al. [18] (experimental mechanistic study); Ciaffi et al. [19] (pilot interventional study; n = 20, 18 completed)	Improvements in pain, mood, and quality of life	Very limited clinical evidence; long-term safety unclear

Polyphenols and Antioxidants

Polyphenol-rich foods, including berries, tea, coffee, chocolate, and olive oil, are important sources of bioactive compounds with potent antioxidant, anti-inflammatory, and neuroprotective properties. These compounds may counteract the effects of reactive oxygen species and reduce oxidative stress, which is increasingly recognised as a contributing factor in the pathophysiology of FM. In addition, polyphenols may influence neuroimmune interactions through modulation of microglial cell activity, thereby potentially reducing central sensitisation and pain perception [38,39].

In addition to dietary sources, antioxidant supplements such as vitamins C and E and N-acetylcysteine have also demonstrated potential therapeutic benefits in FM. These agents may help replenish endogenous antioxidant reserves, including glutathione, and reduce oxidative damage associated with chronic inflammation observed in FM [38,39]. However, the clinical effectiveness of polyphenols and antioxidant interventions may vary because of differences in their bioavailability, intestinal absorption, metabolism by the gut microbiota, and interindividual variability in metabolic responses. Consequently, the magnitude of clinical benefit may differ among patients despite promising experimental findings [40]. Although the available evidence remains preliminary, such nutritional approaches appear promising as adjunctive therapies in the management of FM [38-40].

Coenzyme Q10 (CoQ10)

CoQ10 plays an essential role in mitochondrial energy production by facilitating electron transport within the respiratory chain, thereby contributing to ATP synthesis [41]. In addition, CoQ10 possesses significant antioxidant properties that may help reduce reactive oxygen species and oxidative stress, both of which are implicated in the pathophysiology of FM [41].

In a randomised double-blind placebo-controlled trial, supplementation with CoQ10 at a dose of 300 mg/day for 40 days was associated with significant reductions in FM-related symptoms, including pain, fatigue, and tender points, along with improvements in mitochondrial and inflammatory biomarkers [42]. Earlier clinical observations using the same daily dose for up to three months also reported improvements in clinical symptoms, suggesting that CoQ10 may offer benefit over short- to medium-term supplementation [41]. However, the optimal duration of therapy and long-term efficacy and safety remain uncertain and require confirmation in larger clinical trials [41,42].

Magnesium

Obesity is strongly associated with increased symptom severity in FM [43]. Adipose tissue functions as an active endocrine organ and secretes adipokines, including leptin, as well as pro-inflammatory cytokines such as TNF-α, which may contribute to low-grade systemic inflammation, enhanced pain sensitivity, and metabolic dysfunction. However, FM also exhibits disease-specific inflammatory alterations, including distinct cytokine and chemokine patterns that are associated with pain independently of obesity, suggesting that obesity-related inflammation may amplify, rather than solely drive, the underlying pathophysiology of FM [43,44]. Weight-loss interventions, whether achieved through caloric restriction or adherence to anti-inflammatory dietary patterns, have been associated with reductions in systemic inflammation and improvements in FM-related symptoms [44].

Magnesium supplementation has shown potential benefits in the management of pain and stress in patients with FM. One randomised double-blind clinical trial demonstrated significant reductions in pain intensity and improvements in stress levels following magnesium supplementation. In this study, magnesium was administered as a magnesium chloride technology formulation (ChronoMag®), which was selected because of its favourable absorption characteristics. However, comparative evidence regarding the clinical effectiveness of different magnesium formulations in patients with FM remains limited. Although these benefits were observed primarily in patients with mild-to-moderate stress, findings have not been entirely consistent across studies. Nevertheless, current evidence suggests a possible supportive role for magnesium in FM management [43,44].

Weight Management and Metabolic Health

Obesity is strongly associated with increased symptom severity in FM [44]. Adipose tissue functions as an active endocrine organ and secretes pro-inflammatory cytokines, which may contribute to enhanced pain sensitivity and systemic inflammation. Weight-loss interventions, whether achieved through caloric restriction or adherence to anti-inflammatory dietary patterns, have been associated with reductions in systemic inflammation and improvements in FM-related symptoms [45].

Anti-inflammatory dietary approaches may also promote healthier body composition by enhancing satiety, improving insulin sensitivity, and reducing the consumption of energy-dense processed foods.

The findings of this review suggest that anti-inflammatory dietary approaches may serve a supportive role in the management of FM by targeting key underlying mechanisms, including inflammation, oxidative stress, and alterations in the gut microbiota. Although several dietary patterns have demonstrated potential benefits, the strength and consistency of the available evidence vary considerably. Among the interventions evaluated, Mediterranean and plant-based diets appear to show relatively more consistent improvements in symptom burden and quality of life compared with other dietary approaches [17,29,33].

The management of FM has traditionally relied on pharmacological therapies, including antidepressants, anticonvulsants, and analgesics. Non-pharmacological interventions such as exercise therapy and cognitive behavioural therapy are also commonly incorporated into treatment strategies [1]. Although these approaches may help reduce symptom severity, their effectiveness is often variable and may be associated with adverse effects. In comparison, dietary interventions represent a relatively low-risk strategy that may help support symptom management in patients with FM. Rather than replacing conventional therapies, dietary modifications may be integrated into a multidisciplinary management approach to improve overall clinical outcomes [46].

Current evidence suggests that anti-inflammatory dietary patterns may provide meaningful benefits for individuals with FM, particularly in improving pain, functional status, quality of life, and selected patient-reported outcomes. These findings are consistent with previous systematic reviews and a best-evidence synthesis, which reported potential benefits of dietary interventions such as vegan, hypocaloric, and low-FODMAP diets, while emphasising that the overall certainty of evidence remains low because of methodological limitations and heterogeneity across studies [33,47]. Mechanistic evidence further supports the role of dietary interventions in modulating inflammation, oxidative stress, gut microbiota composition, and mitochondrial function, all of which are implicated in the pathophysiology of FM. However, several important limitations remain, including the limited number of large randomised controlled trials, lack of standardised dietary protocols, reliance on self-reported dietary adherence, and considerable heterogeneity in diagnostic criteria, dietary interventions, and outcome assessment methods, all of which complicate comparisons across studies and preclude firm clinical recommendations [33,47].

Despite these limitations, dietary interventions offer several advantages that are often not achievable with pharmacological therapies alone, including a favourable safety profile, broad accessibility, potential systemic health benefits, and synergistic effects when combined with exercise, sleep hygiene, and psychological interventions. Consequently, dietary counselling should be considered an integral component of FM management within a collaborative and individualised care model, ideally involving registered dietitians with expertise in chronic pain management [46].

## Conclusions

Dietary approaches aimed at reducing inflammation, including Mediterranean, plant-based, low-FODMAP, and elimination diets, appear to provide potential benefits for patients with FM. In addition, supplements such as omega-3 fatty acids, CoQ10, and antioxidants may help target underlying mechanisms, including oxidative stress and mitochondrial dysfunction. These strategies may therefore serve as valuable adjuncts to standard therapeutic approaches, particularly given their relatively favourable safety profile.

However, the available evidence remains limited and somewhat inconsistent, with many studies characterised by small sample sizes, methodological heterogeneity, and variable outcome measures. Consequently, larger and well-designed clinical trials are required before definitive recommendations can be established. Until more robust evidence becomes available, dietary interventions may be considered on an individualised basis as part of a broader multidisciplinary strategy for FM management.
